# Changes in neuronal CycD/Cdk4 activity affect aging, neurodegeneration, and oxidative stress

**DOI:** 10.1111/acel.12376

**Published:** 2015-07-29

**Authors:** Amalia Icreverzi, Aida Flor A de la Cruz, David W Walker, Bruce A Edgar

**Affiliations:** 1 Department of Integrative Biology and Physiology, University of California Los AngelesLos Angeles, CA, 90095, USA; 2 Basic Science Division, Fred Hutchinson Cancer Research CenterSeattle, WA, 98109, USA; 3 German Cancer Research Center (DKFZ) & Center for Molecular Biology Heidelberg (ZMBH)Im Neuenheimer Feld 282, D-69120, Heidelberg, Germany

**Keywords:** aging, mitochondria, neurodegeneration, oxidative stress, superoxide

## Abstract

Mitochondrial dysfunction has been implicated in human diseases, including cancer, and proposed to accelerate aging. The *Drosophila* Cyclin-dependent protein kinase complex cyclin D/cyclin-dependent kinase 4 (CycD/Cdk4) promotes cellular growth by stimulating mitochondrial biogenesis. Here, we examine the neurodegenerative and aging consequences of altering CycD/Cdk4 function in *Drosophila*. We show that pan-neuronal loss or gain of CycD/Cdk4 increases mitochondrial superoxide, oxidative stress markers, and neurodegeneration and decreases lifespan. We find that RNAi-mediated depletion of the mitochondrial transcription factor, Tfam, can abrogate CycD/Cdk4’s detrimental effects on both lifespan and neurodegeneration. This indicates that CycD/Cdk4’s pathological consequences are mediated through altered mitochondrial function and a concomitant increase in reactive oxygen species. In support of this, we demonstrate that CycD/Cdk4 activity levels in the brain affect the expression of a set of ‘oxidative stress’ genes. Our results indicate that the precise regulation of neuronal CycD/Cdk4 activity is important to limit mitochondrial reactive oxygen species production and prevent neurodegeneration.

## Introduction

The relationship between mitochondrial function and aging has been studied extensively. The mutation of several genes involved in mitochondrial biology has been shown to impair mitochondrial function and metabolism and limit lifespan but also disrupts development, behavior, neurological function, and fertility (Hales & Fuller, 1997; Walker *et al*., 2006; Fernandez-Ayala *et al*., 2010). It is striking, however, that studies in both *Caenorhabditis elegans* and *Drosophila* have shown that suppression of genes involved in mitochondrial function can extend lifespan (Lee *et al*., 2003; Copeland *et al*., 2009). These animals with compromised mitochondria displayed enhanced resistance to hydrogen peroxide, suggesting that they have specific modifications in their reactive oxygen species (ROS) pathway (Lee *et al*., 2003). Recent studies in *C. elegans* have demonstrated that altered mitochondrial function in key tissues, such as neurons, is essential for establishing and maintaining a pro-longevity cue systemically (Durieux *et al*., 2011). Moreover, in *Drosophila*, knockdown of electron transport chain (ETC) components with RNAi systemically or in neurons extended lifespan (Copeland *et al*., 2009). Studies on the relationship between longevity and mitochondrial biology do not agree on whether a decrease in mitochondrial function leads to increased or decreased lifespan; rather, the animals’ ability to manage oxidative stress appears to be a strong determinant of lifespan. Indeed, other studies in *Drosophila* have demonstrated a relationship between oxidative stress and lifespan determination (Muller *et al*., 2007).

The aged laboratory animal has several transcriptional, mitochondrial, and metabolic modifications. In *Drosophila*, the aged animal has increased metabolic rates, mtDNA copies, and mtDNA mutations (Melvin *et al*., 2007). Gruenewald *et al*. (2009) determined that a core set of coregulated genes are upregulated in *Drosophila* with old age or by oxidative insults and labeled these genes ‘antioxidant and neuro-protective’. Furthermore, they reported that hyperoxia-induced damage can be a direct cause of brain degeneration and established an experimental setup for measuring neuron survival under oxidative stress (Gruenewald *et al*., 2009). Recent proteomic analysis of mouse brain indicated that the mitochondrial metabolic proteome changes concomitant with age, including many ETC components and Tfam (Stauch *et al*., [Bibr b501]).

The cyclin-dependent protein kinase complex cyclin D/cyclin-dependent kinase 4 (CycD/Cdk4) has been shown to regulate both cellular growth (accumulation of mass) and proliferation (cell cycle progression) in *Drosophila melanogaster* (Datar *et al*., 2000; Buttitta *et al*., 2007). CycD1 also regulates transcriptional pathways involved in metabolism of carbohydrates, lipids, and amino acids (Yu *et al*., 2005). Additionally, many studies have shown that inappropriate activation of CycD/Cdk4 activity leads to disease-associated neuronal death (Greene *et al*., 2007). We have reported that in *Drosophila*, increased CycD/Cdk4 activity leads to increased mitochondrial biogenesis and increased levels and activity of Tfam, the major mitochondrial transcription factor (Icreverzi *et al*., 2012). Additionally, systemic loss or gain of CycD/Cdk4 increases mitochondrial superoxide and decreases lifespan (Icreverzi *et al*., 2012). Here, we show that pan-neuronally expressed CycD/Cdk4 also reduces lifespan, indicating that neuronal tissues may be sensitive to the oxidative stress induced by CycD/Cdk4 activity. Furthermore, we find that neuronal alterations in CycD/Cdk4 activity increase markers of oxidative stress and neurodegeneration. Tellingly, the knockdown of Tfam with RNAi can suppress CycD/Cdk4’s detrimental effects on both lifespan and neuronal death, implying that CycD/Cdk4’s pathological consequences are mediated through its promotion of mitochondrial biogenesis and the concomitant increase in ROS.

## Results

### Neuronal CycD/Cdk4 reduces lifespan

**Fig 1:**
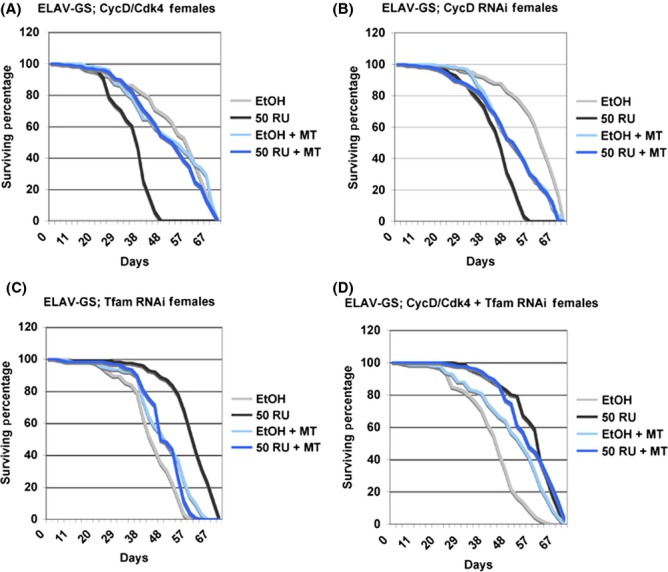
Adult Neuronal CycD/Cdk4 reduces lifespan. Lifespan analysis of flies expressing the indicated genes using the pan-neuronal driver, *ELAV-GeneSwitch*. (A) Adult females expressing *ELAV-GeneSwitch* and CycD/Cdk4 (EtOH vs. RU *P* = 0, EtOH MT vs. RU + MT *P* = 0.133). (B) ELAV-GeneSwitch; CycD RNAi (EtOH vs. RU *P* = 0, EtOH MT vs. RU + MT *P* = 0.788). (C) ELAV-GeneSwitch; Tfam RNAi (EtOH vs. RU *P* = 0, EtOH MT vs. RU + MT *P* = 0.0976). (D) ELAV-GeneSwitch; CycD/Cdk4 + Tfam RNAi (EtOH vs. RU *P* = 0, EtOH MT vs. RU + MT *P* = 0.0101). EtOH: ethanol-fed control. 50 RU: 50 ug mL^−1^ RU-486 GeneSwitch induction. MT, MitoTEMPO.

We previously observed that either loss- or gain-of-CycD/Cdk4 expression decreased mean lifespan and increased mitochondrial superoxide in *Drosophila* (Icreverzi *et al*., 2012). Hyperoxic stress and advanced age both induce neurodegeneration in laboratory animals, indicating that neural tissues may be especially sensitive to oxidative stress (Gruenewald *et al*., 2009). Hence, we used a conditional pan-neuronal driver (*ELAV-GeneSwitch*) to test the consequence of neuronal CycD/Cdk4 overexpression. We used the *ELAV-GeneSwitch* expression method (Yao *et al*., 1993), in which Gal4 is activated by a steroid added to the diet (mifepristone, RU486) to activate UAS-linked gene expression in all neurons in newly eclosed adults. This is advantageous for lifespan studies because effects from genetic variation can be excluded. Pan-neuronal overexpression of CycD/Cdk4 decreased lifespan in both genders (Figs1A and S1C). Paradoxically, neuronal knockdown of CycD via RNAi was also detrimental to lifespan in both genders (Figs1B and S1D).

To determine the effects of neuronal mitochondrial function on lifespan, we expressed RNAi against the mitochondrial transcription factor, *Tfam*. Tfam is necessary for transcription of the mitochondrial genome, which encodes 13 essential subunits of the inner mitochondrial membrane respiratory complexes, I, III, IV, and V, as well as tRNAs and rRNAs. Neuronal knockdown of *Tfam* with RNAi strongly prolonged lifespan in females (Fig.1C) although the effect was not significant in males (Fig. S1E). This is similar to the longevity promoting effects reported by Durieux *et al*. (2011)*,* who expressed RNAi against an ETC component neuronally. Other studies in *Drosophila* have indicated that systemic or neuronal RNAi against ETC components also prolongs lifespan (Copeland *et al*., 2009). We believe that Tfam RNAi indirectly targets core mitochondrially encoded ETC components to induce levels of mitokines (mitochondrial stress signals) that promote longevity.

To test whether the lifespan effects of CycD were due to its effects on mitochondria, we performed epistasis tests in which Tfam RNAi was co-expressed along with CycD/Cdk4. The expression of Tfam RNAi in males co-expressing CycD/Cdk4 completely rescued their lifespan deficits (Fig. S1F, *P* = 0.341 compared to controls). Yet more striking, females expressing both CycD/Cdk4 and Tfam RNAi still had statistically increased lifespans (*P* = 0.032, Fig.1D).

As CycD and its cognate kinase partner, Cdk4, are both classified as core cell cycle components, one might expect them to be absent from postmitotic cells. Hence, we measured the levels of CycD mRNA in adult brains to determine whether there was any postmitotic expression. We found that *cycD* mutant heads had more than a 20-fold decrease in CycD mRNA (compared to control heads) indicating endogenous CycD mRNA in WT adult brains (Fig. S2). Furthermore, using the *ELAV-GeneSwitch* driver, we are able to knockdown this endogenous message using CycD RNAi (*P* < 0.02, Fig. S2). Indeed, Flybase reports that CycD message is found at ‘low expression levels’ in the adult head, eye, and brain (flybase.org). Similarly, Cdk4 message is found at ‘low expression levels’ in the adult head and eye (flybase.org). The presence of endogenous CycD and Cdk4 expression in the adult head, together with the negative effects of CycD/Cdk4 overexpression or knockdown in neurons, suggests that the precise control of CycD/Cdk4 activity is important for normal neural function.

To determine whether the expression of CycD/Cdk4 expression was detrimental in neural cell types other than differentiated neurons, we used the *worniu-Gal4* driver, which is expressed in neuroblasts and ganglion mother cells of the brain hemispheres and ventral ganglions (Ashraf *et al*., 2004). CycD/Cdk4 expression driven by *worniu-Gal4* was not larval lethal and did not influence lifespan, indicating that excess CycD/Cdk4 lifespan-reduction effect is adult neuron specific and limited to cells that express *ELAV-GeneSwitch* (Fig. S3).

### Loss or gain of CycD/Cdk4 increases mitochondrial ROS

**Fig 2:**
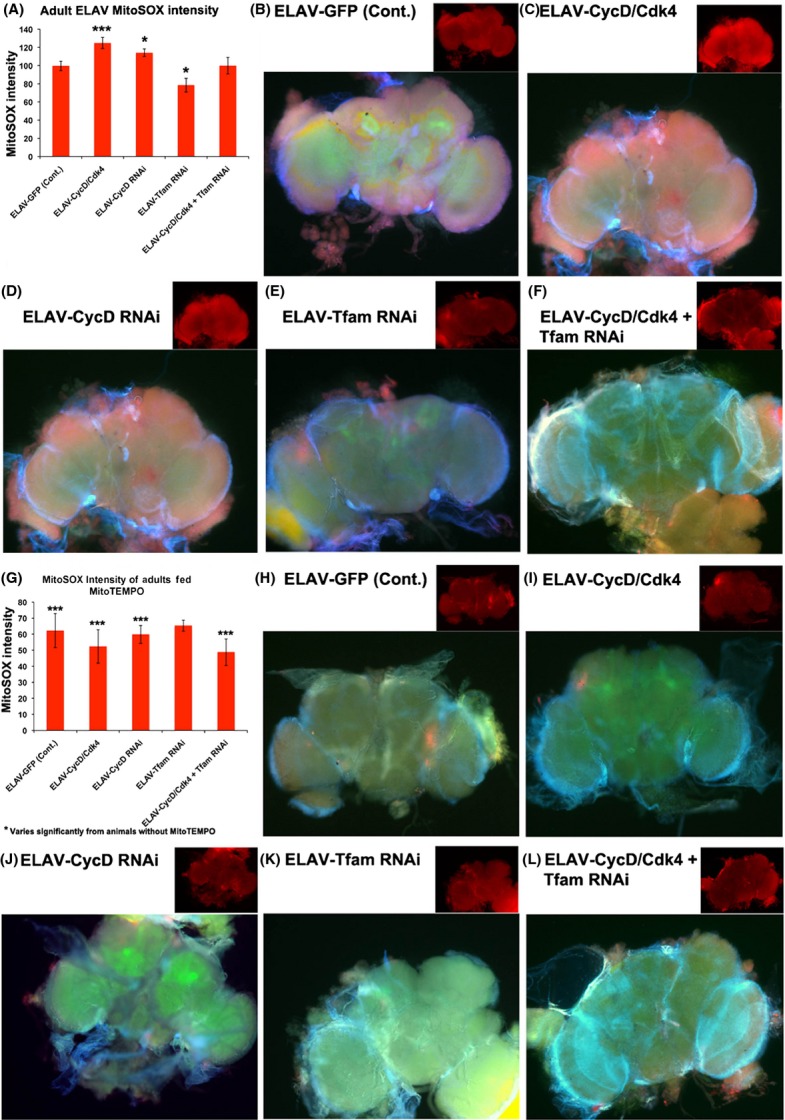
Neuronal loss or gain of CycD/Cdk4 increases superoxide production. MitoSOX staining in central nervous system cells expressing the indicated transgenes under *ELAV-Gal4* control; cells are marked with UAS-GFP in 14-day-old adults. (A) Graph of quantification of MitoSOX intensity of ELAV-GFP, *P < 0.05, ***P < 0.001. (B) ELAV-GFP (control), (C) CycD/Cdk4, (D) CycD RNAi, (E) Tfam RNAi, (F) CycD/Cdk4 + Tfam RNAi. (G) Graph of quantification of MitoSOX intensity of ELAV-GFP on MitoTEMPO (H) ELAV-GFP (control) on MitoTEMPO, (I) CycD/Cdk4, (J) CycD RNAi, (K) Tfam RNAi, (L) CycD/Cdk4 + Tfam RNAi. *** varies significantly from animals without MitoTEMPO.

Our results suggest that excess or lack of neuronal CycD/Cdk4 may lead to an ‘aged’ phenotype. Aging has been affiliated with increased ROS production (Chakravarti & Chakravarti, 2007). To investigate whether excess or loss of CycD/Cdk4 led to increased ROS, we utilized Molecular Probes’ MitoSOX reagent. MitoSOX is a mitochondrial-targeted superoxide indicator and is a selective detector of the superoxide anion generated as a byproduct of mitochondrial OXPHOS (Batandier *et al*., 2002). We found that excess neuronal CycD/Cdk4 increased MitoSOX intensity, as did CycD RNAi, by 25% and 15%, respectively (Fig.2). To specifically identify the source of the superoxide, we used a cytoplasmic superoxide indicator, dihydroethidium (DHE) (Bahadorani *et al*., 2010). *ELAV-*CycD/Cdk4 expression did not alter DHE staining in central nervous system (CNS) cells, indicating that mitochondria are the main source of superoxide in these cells (Fig. S4). Knockdown of CycD with RNAi decreased the cytoplasmic levels of DHE, again indicating exclusive mitochondrial localization of superoxide generation in CNS cells with CycD knockdown via RNAi (Fig. S4).

To further test the role of mitochondria in superoxide production, we inhibited mitochondrial function with RNAi against Tfam and assessed the MitoSOX signal. Tfam RNAi reduced the CNS MitoSOX signal by 20%, and CycD/Cdk4-expressing brains with additional Tfam RNAi had MitoSOX levels comparable to controls (Fig.2).

In addition, we reduced mitochondrial superoxide in CycD/Cdk4 and CycD RNAi animals with a mitochondrially targeted superoxide dismutase (SOD) mimetic added to their food, MitoTEMPO (MT). Indeed, when we measured the MitoSOX levels of animals fed MT, we found significantly reduced levels of MitoSOX in all genotypes except for Tfam RNAi-expressing animals (Fig.2). Notably, we saw a significant rescue of CycD/Cdk4-overexpressing or CycD RNAi-expressing flies’ lifespans in both males and females (Figs1 and S1). However, MT did not extend the lifespan extension of Tfam RNAi-expressing animals, but rather shortened it (Figs1C and S1E). We hypothesize that MT neutralized the excess mitochondrial ROS in CycD/Cdk4 or CycD RNAi-expressing cells and thereby rescued those animals’ lifespans. But the neutralization of mitochondrial superoxide in Tfam RNAi-expressing animals did not improve their lifespans, suggesting that endogenous mitochondrial ROS may be an important signaling molecule and that precise levels of mitochondrial ROS need to be maintained for healthy aging (Ristow & Zarse, 2010).

### Neuronal CycD/Cdk4 promotes protein carbonyl modification

**Fig 3:**
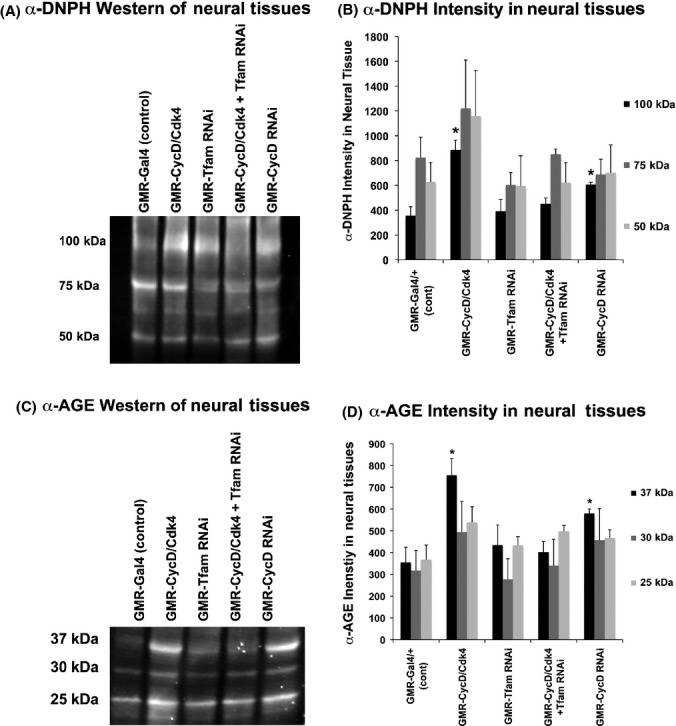
Reactive oxygen species induced protein carbonyls and oxidative stress-dependent advanced glycation end products (AGEs). (A) Western blot incubated with the protein carbonyl detection with genotypes listed above every lane. Four male heads expressing *GMR-Gal4* for 7 days were used for each sample. (B) Graph quantifying the intensity of three separate blots at several bands, * indicates *P* < 0.05. (C) Image of Western blot incubated with α-AGE antibodies, with genotypes listed above each lane. Four male heads expressing *GMR-Gal4* for 7 days were used for each sample. (D) Graph quantifying the intensity of three separate blots at several bands. * indicates *P* < 0.05.

To study the functional consequence of increased superoxide in CycD/Cdk4-overexpressing brains, we next analyzed protein carbonyl modification. Several studies have demonstrated that hyperoxia-induced damage is a direct cause of brain degeneration in *Drosophila*, and report that oxidative stress and aging is associated with increased protein carbonylation (Botella *et al*., 2004; Gruenewald *et al*., 2009; Jacobson *et al*., 2010). Protein cabonyl groups occur as a result of oxidative damage, during aging and certain disease states; carbonyl groups are chemically stable and serve as markers of oxidative stress (Chakravarti & Chakravarti, 2007). Using the OxiSelect Protein Carbonyl Immunoblot Kit, we analyzed carbonyl-modified proteins in flies that overexpressed genes of interest with the *GMR-Gal4* promoter, which induces neuronal (photoreceptor)-specific expression late in pupal development and also in the adult eye (Tao Wang, personal communication). Using the carbonyl-DNPH detection kit, adult heads with *GMR-Gal4* expression showed relatively high levels of carbonyl modification of proteins of 100, 75 and 50 kDa (at 7 days overexpression, DO; Fig.3). Both overexpressed CycD/Cdk4 and CycD RNAi significantly increased carbonyl-modified proteins of about 100 kDa size, whereas the protein levels at those sizes appear unchanged via Coomassie stain (Figs3 and S6). The addition of Tfam RNAi to CycD/Cdk4 rescued the predominant carbonyl-modified protein species at 100 kDa, indicating that CycD/Cdk4 requires Tfam to induce carbonyl-modified proteins (Fig.3). These results could also be due to different proteins being predominantly expressed in the ‘CycD/Cdk4-aging’ fly eye, or to carbonyl-modified proteins being either preferentially degraded or enzymatically remedied by the carbonyl reductase, *Drosophila* sniffer (Botella *et al*., 2004). More tests need to be performed in order to determine precisely how excess neuronal CycD/Cdk4 promotes the carbonyl modification of specific proteins.

Additionally, we attempted to reduce and neutralize mitochondrial oxidative stress in GMR-CycD/Cdk4- and CycD RNAi-expressing animals with MT. We saw a significant rescue of CycD/Cdk4 and CycD RNAi carbonyl modification and an overall dampening of carbonyl-modified proteins in animals fed MT (Fig. S5). We therefore conclude that reducing mitochondrial superoxide is sufficient to lessen the oxidative stress that leads to protein carbonyl modification in cells with excess or insufficient CycD/Cdk4 activity (Fig. S5).

### Neuronal CycD/Cdk4 promotes advanced glycation end products

To further assess oxidative damage, we next assayed the accumulation of advanced glycation end products (AGEs). In conditions of high ROS, cellular proteins can be damaged directly by oxidation, but also indirectly by nonenzymatic glycation, when reducing sugars become chemically attached to proteins and irreversibly oxidized to form AGEs (Chakravarti & Chakravarti, 2007). High AGE levels are associated with increased mortality rates in flies and thus are classified as biomarkers of aging and oxidative stress damage in *Drosophila* (Chakravarti & Chakravarti, 2007; Jacobson *et al*., 2010).

Using an α-AGE antibody, we analyzed AGE levels in flies that overexpressed transgenes with the *GMR-Gal4* promoter. In adult neural tissues expressing UAS-CycD/Cdk4 or UAS-CycD RNAi under *GMR-Gal4* control, we detected increased AGEs of 37, 30, and 25 kDa, whereas the protein levels at those sizes appeared unchanged via Coomassie stain (at 7 days overexpression, DO; Figs3C,D and S6). Either CycD/Cdk4 overexpression or CycD knockdown with RNAi significantly increased AGEs of around 37 kDa. Importantly, depletion of Tfam with RNAi suppressed the increase in AGE-modified proteins in the CycD/Cdk4-overexpressing animals, further indicating that CycD/Cdk4 promotes oxidative stress via the mitochondria (Figs3 and S6). Similarly, we saw a significant suppression in the CycD-dependent AGE modification in animals fed MT (Fig. S5). We therefore conclude that reducing mitochondrial superoxide via MT antioxidant is sufficient to lessen the oxidative stress that leads to protein AGE modification in cells with excess or insufficient CycD/Cdk4 activity.

### Neuronal gain or loss of CycD/Cdk4 results in cell death

**Fig 4:**
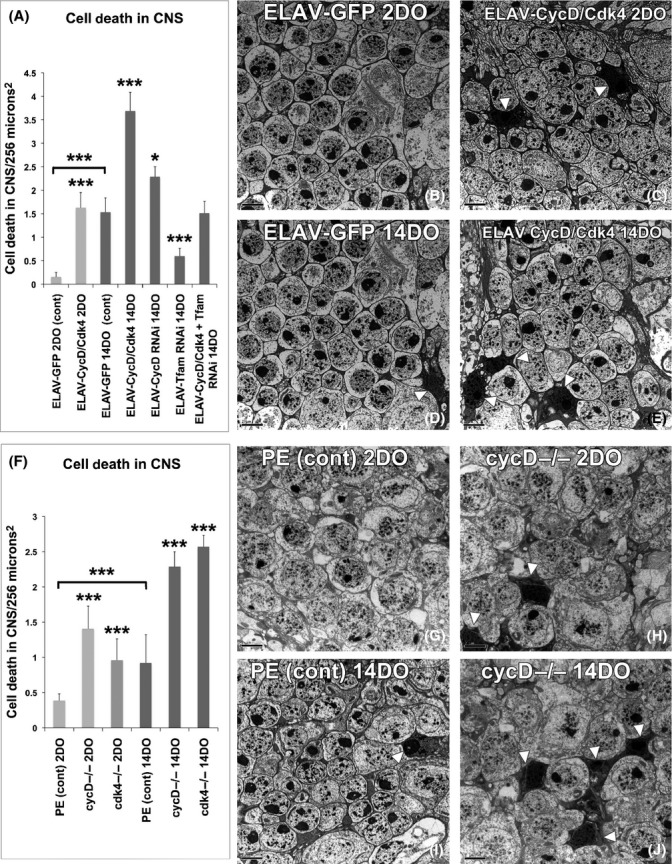
Neuronal loss or gain of CycD/Cdk4 induces cell death. (A) Graph quantifying death in adult laminar neurons from the following genotypes (* indicates *P* < 0.05, ***P < 0.001). (B) Adult male central nervous system (CNS) expressing ELAV-GFP/+ for 2 days, (C) Adult male CNS expressing ELAV-CycD/Cdk4 for 2 days (white arrow heads pointed at dead cells, which stain denser), (D) adult male expressing ELAV-GFP/+ for 14 days, (E) ELAV-CycD/Cdk4 for 14 days, (F) graph quantifying death in laminar neurons in mutants (***P < 0.001). (G) Precise excision (PE) control at 2 days, (H) *cycD* mutants at 2 days, (I) PE control at 14 days, (J) cycD mutants at 14 days; other images in Supporting Information.

To determine whether neuronal CycD/Cdk4 expression was responsible for inducing neurodegeneration and cell death *in vivo,* we analyzed flies via electron microscopy. Using this approach, Gruenewald *et al*. (2009) demonstrated that hyperoxia-induced damage causes brain degeneration in *Drosophila,* and established an experimental setup for measuring neuron survival under oxidative stress. They determined that oxidative stress caused specific ultrastructural alterations in certain brain regions, namely hyperoxia-induced vacuolization and the appearance of dead cells in distal sections of the optic lobe (Gruenewald *et al*., 2009). We analyzed the same adult CNS regions, specifically the laminar neurons between the retina and the optic lobe, scoring for the frequency of electron-dense dead cells. In controls, the expression of *UAS-GFP* under *ELAV-Gal4* control for 2 days (DO) resulted in ∼0.1 cell deaths per laminar section, while the overexpression of CycD/Cdk4 significantly increased the number to 2 cell deaths per 256 μm^2^ (Fig.4). Older adults with *ELAV-GFP* (control) at 14 days had increases in cell death per laminar section (to 1.5), which was significantly different than the cell death index in young flies’ brains, indicating that these laminar neurons become susceptible to cell death as animals age (Gruenewald *et al*., 2009). The prolonged overexpression of CycD/Cdk4 in adult brains with *ELAV-Gal4* leads to an even larger increase in cell death of laminar neurons at 14 days (to 4), indicating that not only are these neurons vulnerable to age-related neuronal death but the addition of excess CycD/Cdk4 enhances this progressive cell death. The depletion of CycD with RNAi in neurons led to modest but significant increases in the neuronal death index (Figs4 and S6). The depletion of Tfam with RNAi in neurons led to a significant reduction in age-related neuronal death. As expected, the depletion of Tfam in cells co-expressing CycD/Cdk4 rescued their cell death index to control levels, suggesting that the CycD/Cdk4-dependent increases in mitochondrial superoxide (Fig.2) may influence neuronal cell death rates.

To confirm these RNAi results, we investigated the same adult CNS regions responsive to hyperoxic stress, in *cycD* or *cdk4* mutants (Gruenewald *et al*., 2009). We found that in young mutant adults, the loss of either *cycD* or *cdk4* increased neuronal cell death two- to threefold (Figs4 and S7). Older control animals had increased cell death relative to the younger controls, again demonstrating that these neurons are susceptible to age-related cell death. In older *cycD* or *cdk4* mutants, we saw more than a doubling in neuronal cell death relative to older controls. Consistent with our other results, this suggests that endogenous CycD/Cdk4 activity is crucial for optimal adult neuronal survival (Figs4 and S7).

Our laboratory has previously demonstrated that the induction of autophagy suppresses cell death in *Drosophila* neurodegenerative disease (Wang *et al*., 2009). Using the same methods, we investigated whether excess or insufficient CycD/Cdk4 activity is causing cell death by suppression of autophagy (Wang *et al*., 2009). We found that neither GMR-CycD/Cdk4 nor GMR-CycD RNAi induces neurodegeneration via suppression of autophagy in either young or aged animals (Fig. S9).

### MT suppresses neuronal death caused by either gain or loss of CycD/Cdk4

The loss- or gain-of-CycD/Cdk4 function in adult neurons affects neuronal cell death rates and directly correlates with both our lifespan and MitoSOX results. This suggests that mitochondrial superoxide may be responsible for these neurons’ death. To test this, we fed animals MT to neutralize their cells’ mitochondrial superoxide and assessed cell death in the CNS laminar neurons via electron microscopy. We found that these animals’ CNSs had similar levels of neuronal cell death as MT-fed controls, irrespective of genotype. Thus, laminar neuron cell death can be suppressed by mitochondrial superoxide neutralization. Similarly, the lifespans of these animals were also restored to control lengths by MT (Figs1 and S1).

### Excess CycD/Cdk4 upregulates hyperoxia-responsive transcripts

Aged laboratory animals have several transcriptional, mitochondrial, and metabolic changes (Melvin *et al*., 2007). Gruenewald *et al*. (2009) identified a set of transcripts upregulated in *Drosophila* by old age, or by oxidative stress induced by either mitochondrial poisons (paraquat) or hyperoxia (100% O_2_), and designated these mRNAs ‘antioxidant neuroprotective’ transcripts. We found that a statistically similar set of ‘oxidative stress’ genes was upregulated by CycD/Cdk4 activity (Fig.6 and Table S1). Hyperoxia-responsive (antioxidant) transcripts were statistically upregulated in animals overexpressing CycD/Cdk4 for 16 h, suggesting a relatively direct mechanism (*P* < 1.57e^−05^, Fig.6, Table S1; Gruenewald *et al*., 2009). To determine whether the CycD/Cdk4-overexpressing animals upregulated genes involved in ROS neutralization, we looked for an increase in the transcripts encoding the SOD, catalase (CAT), and glutathione reductase (GSH) enzymes, and we found increases in both CAT and GSH (Table S1; Gruenewald *et al*., 2009).

When we compared transcripts that were upregulated in both *cycD* mutants and hyperoxia-treated animals, we found no significant similarities. Rather, *cycD* mutants significantly downregulated many transcripts that were upregulated by hyperoxia (Fig.6, Table S2). Furthermore, *cycD* mutants had significant repression of *catalase* and *peroxiredoxin* transcripts, both enzymes involved in ROS neutralization. This suggests that *cycD* mutants may transcriptionally repress the oxidative stress response (Table S2).

To study the functional relevance of neuronal CycD/Cdk4-driven gene expression, we used the *ELAV-Gal4* driver to further analyze hyperoxia-responsive transcripts (Gruenewald *et al*., 2009). We focused on transcripts that had been verified by Gruenewald *et al*. (2009) as being functionally over-represented, including those encoding chaperones (Hsp22), detoxification/antioxidant (Cyp6d2, Cyp309a1), oxidoreductase (*sni*-carbonyl reductase), and UDP-glucuronosyl/UDP-glucosyltransferase (CG5999). *ELAV-Gal4-*driven CycD/Cdk4 overexpression increased the transcripts of all these genes and most significantly amplified the transcripts encoding Cyp309a1 (an antioxidant) and Hsp22 (a mitochondrial chaperone) (Fig.6). This swift and direct transcriptional upregulation of hyperoxia-responsive genes indicates that CycD/Cdk4 activity might be important for adult neuronal survival in the face of oxidative stress (Fig.6, Tables S1 and S3).

Certain transcripts are also downregulated by hyperoxia exposure, and flies mutant for those genes fare better in prolonged hyperoxia (Zhao *et al*., 2010). Specifically, mutation of tropomyosin 1, glycerol 3 phosphate dehydrogenase, or CG33129 confers tolerance to severe hyperoxia (Zhao *et al*., 2010). In our laboratory, overexpression of CycD/Cdk4 significantly repressed three of those genes and many other transcripts that are downregulated in hyperoxia-adapted flies to a similar degree (*P* < 2.74e^−15^, Table S3; Zhao *et al*., 2010).

Overall, flies overexpressing CycD/Cdk4 appeared to have a significantly similar transcriptional profile to aged or oxidatively stressed animals. This suggests that along with upregulation of growth and mitobiogenesis, CycD/Cdk4 upregulates transcripts important for managing oxidative stress, a byproduct of mitochondrial metabolism (Datar *et al*., 2000). *cycD* mutants have an inverse hyperoxia transcriptional profile, and they significantly upregulate transcripts that are downregulated by hyperoxia-adapted flies (Table S4, *P* < 1.811e^−07^; Zhao *et al*., 2010).

## Discussion

In this study, we show that loss- or gain-of-CycD/Cdk4 function influences mitochondrial superoxide production in neurons, neurodegeneration, and lifespan. Both the lifespan deficits and increases in mitochondrial superoxide could be suppressed by RNAi against Tfam or feeding MT, a mitochondrial (SOD) mimetic, indicating that excess mitochondrial superoxide was among most detrimental consequence of neuronal excess CycD/Cdk4 (Figs S3 and S4). When we analyzed the transcriptional profiles of animals overexpressing CycD/Cdk4, we found that they upregulate a significant set of transcripts that are also induced by hyperoxia treatment (100% O_2_) or old age (Gruenewald *et al*., 2009). Sixteen hours of CycD/Cdk4 overexpression induced transcription of genes involved in ROS detoxification and antioxidation, generally to the same degree as oxidation stressors including hyperoxia, old age, and mitochondrial poisons (Gruenewald *et al*., 2009). CycD/Cdk4 activity also induced transcripts encoding glutathione peroxidase (GSH) and catalase (CAT), two enzymes directly involved in the neutralization of superoxide anion (Table S1; Gruenewald *et al*., 2009). Furthermore, CycD/Cdk4 also repressed transcripts shown to be downregulated by sustained hyperoxia-adapted animals, oftentimes suppressing transcripts to the same degree as reported for hyperoxia-adapted animals (Zhao *et al*., 2010).

Pro-oxidative stressors such as 100% O_2_, old age, and mitochondrial poisons have neurodegenerative effects in *Drosophila* (Botella *et al*., 2004; Gruenewald *et al*., 2009). One particular region of the CNS that seems particularly sensitive to pro-oxidant stress is the laminar neurons, which lie in between the retina and the optic lobe in the adult *Drosophila* brain (Botella *et al*., 2004; Gruenewald *et al*., 2009). This region of the brain may be susceptible to oxidative damage as laminar neurons relay the signals from the fly eye to the optic lobe, an especially active brain region of the animal when it is exposed to light stimuli. These laminar neurons are very metabolically active and grow with increased visual stimuli (Barth *et al*., 2010). When we investigated the laminar neurons of flies overexpressing pan-neuronal CycD/Cdk4, we found that there was a progressive age-dependent increase in the number of cell deaths per laminar region (Fig.4). Knockdown of Tfam with RNAi could abrogate CycD/Cdk4’s detrimental effects in the laminar neurons, indicating that cell death caused by excess CycD is likely a result of its ability to stimulate mitobiogenesis. As these neurons were also susceptible to age-related cell death in *cycD* and *cdk4* mutant brains, we consider them to be extremely sensitive to the level of endogenous CycD/Cdk4 activity where its main action may be to promote balanced mitobiogenesis. Interestingly, ample data on the relationship between CycD/Cdk4 activity and neuronal health and development have already been generated in mammalian systems. Firstly, CycD is important for embryonic neurogenesis. CycD1-deficient mice show developmental neurological abnormalities, and CycD2-deficient animals develop cerebellar defects (Kozar *et al*., 2004). Secondly, mammalian CycD2 has been shown to have a critical role in adult neurogenesis in the hippocampus and in the olfactory bulb (Kowalczyk *et al*., 2004). Lastly, CycD/Cdk4 activity is upregulated by adult neurons exposed to oxidative stress, in both mammals and *Drosophila* (Nguyen *et al*., 2003, 2002).

**Fig 5:**
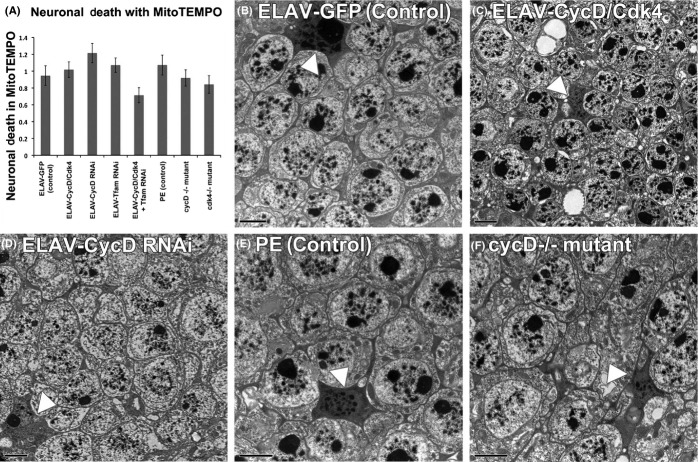
MitoTEMPO prevents neuronal death in gain and loss of CycD. (A) Graph displaying cell death in laminar neurons in adults fed MitoTEMPO for 14 days, (B) adult males expressing ELAV-GFP/+ for 14 days (white arrow heads pointed at dead cells, which stain denser), (C) adult males expressing ELAV-CycD/Cdk4 for 14 days, (D) adult males expressing ELAV-CycD RNAi for 14 days, (E) PE control at 14 days, (F) cycD mutants at 14 days; other images in Supporting Information.

**Fig 6:**
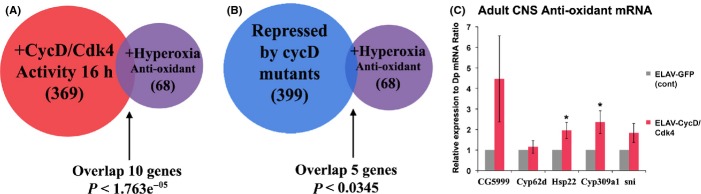
CycD/Cdk4 overexpression affects transcriptional profiles similar to hyperoxia. cDNA microarrays were used to analyze CycD/Cdk4 overexpression and compared with the published data following hyperoxia (Gruenewald *et al*., 2009; Icreverzi *et al*., 2012). (A) Comparison of gene sets following CycD/Cdk4 overexpression with hyperoxia treatment reveals significant overlap, (B) comparison of gene sets from *cycD* mutants repressed transcripts with hyperoxia treatment reveals significant overlap, (C) transcript analysis confirming that hyperoxia-responsive transcripts are present in ELAV-CycD/Cdk4 heads (Gruenewald *et al*., 2009). *p < 0.05.

Previously, based on similar results obtained in different cell types, we suggested that both loss- or gain-of-CycD/Cdk4 activity can lead to an imbalance in mitobiogenesis, resulting in inefficient OXPHOS coupling and increased superoxide production, and we also showed that Tfam RNAi ameliorated these detrimental effects (Icreverzi *et al*., 2012). We demonstrate here that neuronal loss or gain of CycD/Cdk4 has detrimental effects on lifespan, mitochondrial superoxide production, and neurodegeneration (Figs1, 2 and 4). We can suppress many of the detrimental effects of CycD/Cdk4 overexpression by reducing its mitobiogenesis capacity (with the addition of Tfam RNAi) or neutralizing the excess mitochondrial superoxide with MT. We hypothesize that any imbalance in mitobiogenesis leads to disproportionate OXPHOS and increased ROS. This increased ROS leads to the increase in cellular damage (as seen with increased AGE or carbonyl-modified proteins), increased neuronal death, and ultimately organismal death (Figs 1–4). Given that MT supplementation could prevent both phenotypes, we conclude that neuronal and organismal death seen with both loss- or gain-of-CycD/Cdk4 activity are due to excess mitochondrial oxidative stress (Figs1 and 5). Indeed, other studies have shown that supplemental TEMPO can restore redox homeostasis and reduces advanced oxidation protein products (Aksu *et al*., 2014). Our results also suggest that increases in oxidative stress-responsive transcripts are not sufficient to neutralize all the damage caused by excessive oxidative stress in neuronal CycD/Cdk4-overexpressing animals (Figs2, 3 and 6). Nevertheless, these gene expression changes are a further indication of the loss of oxidative homeostasis in these animals. Further studies of the roles of CycD/Cdk4 and TFAM in oxidative homeostasis in mice and man should prove interesting.

## Experimental procedures

### Drosophila genetics

The *Elav-Gal4* driver was utilized for MitoSOX, DHE, mRNA, and neurodegeneration experiments, the *ELAV-GeneSwitch* driver was used in the lifespan analyses, and the *GMR-Gal4* driver was used for Westerns blotting and autophagy experiments. ELAV-GAL4 expresses in all differentiated neurons, whereas GMR-Gal4 expresses in differentiated photoreceptors of the eye. Female parents were controlled among all experiments.

### Lifespan

Adult flies were collected within 16 h of enclosure, mated for 48 h, and then segregated by gender. Flies were assayed for viability and transferred to fresh vials every 2 days as described by Rera *et al*. (2011). Genes were induced via feeding 50 ug of mifepristone (RU-486) per mL of food vials contained 25 adults, with five vials per condition.

### MitoSOX

Fourteen-day-old *ELAV-Gal4*, UAS-GFP/UAS-X adult brains were dissected in PBS, incubated at RT with 5 μm MitoSOX (Molecular Probes) in DMSO for 10 min. Tissues were washed twice in PBS, and fixation was then performed in 8% paraformaldehyde (PFA) in PBS for 10 min with DNA stain Hoechst 33258. Tissues were washed 2 ×  in PBS and mounted with Vectashield (Vector Labs).

### MitoSOX intensity ratios

Twenty images from ELAV-Gal4, UAS-GFP (ELAV-GFP), and UAS-X CNS from adults incubated with MitoSOX were analyzed in ImageJ program for mean pixel intensity of signal in red channel. Images were taken on a Nikon Ti microscope using a Roper Coolsnap HQ2 CCD, using equal exposure times across all samples for each channel. Fluorescence was measured in the Red (MitoSOX) and Green (GFP) channels. Briefly, images were separated into color channels, and CNS expressing ELAV-GFP and UAS-X was measured for mean pixel intensity of the red signal which was quantified in the red channel. Intensity of signal was represented as intensity of signal in ELAV-GFP and UAS-X CNS.

### Electron microscopy

Six heads from 2- and 14-day-old ELAV-Gal4, UAS-GFP/UAS-X, and mutant males were fixed overnight at 4°C in 2% PFA plus 1% glutaraldehyde, postfixed in 1% osmium tetroxide (OsO_4_) at RT, dehydrated in an ethanol series, and embedded in Epon 812. Ultrathin sections (80 nm) were examined with JEOL 1230 transmission electron microscope at 100 kV (as in Wang *et al*., 2009). Hundred micrographs from each genotype were taken at 1000× magnification.

### Quantification of EM micrographs

Ten micrographs from each brain lobe per animal were analyzed from six males of genotype and age listed. A total of 120 total micrographs per genotype of 1000× magnification were analyzed to quantify cell death (darker stained, ‘exploded’ dead cells were counted per image view which measured 256 μm^2^).

### Western blotting detection of protein carbonyls

Four *GMR-Gal4*, UAS-X, fly heads (20 μg total protein) were mixed with 15 μL of 2× sample buffer. Following incubation at 90 °C for 10 min, samples were loaded onto a Bio-Rad 4–12% Bis-Tris minigel. Electrophoresis was performed at 120 V for 90 m. Proteins were electro-transferred to PVDF membrane for 1 h. The membrane was washed in 100% methanol for 15 s and allowed to dry at RT for 5 min. The membrane was equilibrated in Tris-buffered saline (TBS) containing 20% methanol for 5 min and washed 2× in 2N HCl for 5 min. The membrane was incubated with DNPH solution for 10 min at RT, then washed 3× in 2N HCl, 5 min each time, and then washed in 100% methanol 5×, 5 min each time. The membrane was then blocked for 2 h with 5% nonfat milk in TBST at RT. Following three rinses with TBST, the membrane was incubated with rabbit α-DNP antibody (diluted 1:1000 in TBST, CellBioLabs OxiSelect Kit, San Diego, CA USA) overnight at 4 °C and then stained with goat anti-rabbit IgG (diluted 1:20 000 in TBST; Odyssey) for 1 h at RT. Bands were visualized with rabbit-specific secondary antibody and scanned for quantification. The membranes were then stained with Coomassie blue, a general protein-staining dye, after Western blotting to visualize the major proteins in fly heads.

### Western blotting detection of AGE-modified proteins

Four *GMR-Gal4*, UAS-X, fly heads (20 μg total protein) were mixed with 15 μL of 2× sample buffer. Following incubation at 90 °C for 5 min, samples were loaded onto a Bio-Rad 4–12% minigel. Electrophoresis was performed at 120 V for 90 m. Proteins were electro-transferred to NC membrane in transfer buffer at 100 V, 4 °C for 1 h. The membrane was washed twice in water and then blocked for 2 h with blocking buffer at RT. Following three rinses with TBST, the membrane was incubated with rabbit α-AGE polyclonal antibody (Abcam, Cambridge, MA, USA), goat anti-rabbit IgG (diluted 1:40 000 in blocking buffer). Bands were visualized and analyzed as described above.

### Quantification of Western blots

Western membrane blots incubated with fluorescent secondary antibodies were visualized with Li-Cor scanning bed. The mean pixel intensity of the three predominant protein bands was quantified in ImageJ software. The membranes were poststained with Coomassie Blue, and in the area of interest, they showed no loading difference between the samples.

### Significance of overlap

Transcripts that were determined to be significantly up/downregulated by SAM analysis were enumerated and compared to gene sets upregulated in either CycD/Cdk4 overexpression for 16 h, repressed in *cycD* mutants, or upregulated by hyperoxia treatments (Gruenewald *et al*., 2009). The cumulative hypergeometric probability provided by the following Web site determined the statistical significance of the overlap between two groups of genes: http://stattrek.com/Tables/Hypergeometric.aspx.

### mRNA Quantitative RT–PCR

Total RNA was purified from four male heads expressing *ELAV-Gal4*/UAS-X using RNeasy (Qiagen, Valencia, CA, USA). cDNA was synthesized using random hexamers and reverse-transcribed with iScript (Bio-Rad, Hercules, CA, USA) according to the manufacturer’s protocol. RT–PCR was performed by monitoring in real time the increase in fluorescence of the SYBR Green dye (Bio-Rad). Transcript levels were determined using the log_2_ of the Δ between the Ct (cycle threshold) values between the hyperoxia and Dp genes (primers listed in Appendix S1); experiments were carried out in triplicate.

### cDNA microarrays

Using *hs-Flp Act-Gal4* driver to induce CycD/Cdk4 for 16 h in 72AED larvae, we extracted total RNA 16 h later, synthesized cDNA, labeled with Cy5 or Cy3, and hybridized to Drosophila-specific 13K-cDNA microarrays; data also reported in Icreverzi *et al*. (2012). All significantly upregulated transcripts, as determined by significant analysis program (SAM), were compared to Gruenewald *et al*.’s (2009) core set of hyperoxia-responsive genes.

## Supporting Information

**Appendix S1** Fly stocks and UAS transgenes. **Fig. S1** Adult Male ELAV-GS Lifespans and ELAV-GS Female Mortality Rates. **Fig. S2** Adult heads expression levels of CycD and Tfam mRNA. **Fig. S3***Worn-Gal4* loss or gain of CycD/Cdk4 does not influence lifespan. **Fig. S4** DHE Superoxide indicator in *ELAV-GFP* CNS. **Fig. S5** Protein carbonyls and oxidative stress-dependent AGEs eliminated by feeding MitoTEMPO antioxidant. Mutants show no changes in modified protein carbonyls and oxidative stress-dependent AGEs. **Fig. S6** Commassie staining of Westerns stained with DNPH or AGE. **Fig. S7** Neuronal loss or gain of CycD/Cdk4 induces cell death. **Fig. S8** MitoTEMPO prevents neuronal death in loss or gain of CycD/Cdk4. **Fig. S9** Loss or gain of CycD/Cdk4 does not repress autophagy. **Table S1** CycD/Cdk4 overexpression resembles Hyperoxia. **Table S2** cycD mutants repress transcripts upregulated by Hyperoxia. **Table S3** CycD/Cdk4 overexpression suppressed similar transcripts as Hyperoxia treatment. **Table S4** cycD mutants upregulate transcripts suppressed by Hyperoxia treatment.
