# Supplementary material for: Changes in neuronal CycD/Cdk4 activity affect aging, neurodegeneration, and oxidative stress
Source: Aging Cell. 2015 Jul 29;14(5):896–906. doi: 10.1111/acel.12376 (PMC4568977; doi:10.1111/acel.12376)
Supplement: Supplementary file 1 [file acel0014-0896-sd1.doc]

Changes in CycD/Cdk4 affects oxidative stress, neurodegeneration, and lifespan

Amalia Icreverzi, Aida Flor A. De La Cruz, and Bruce A. Edgar

Supporting Information:

Figure S1. Adult Male ELAV-GS Lifespans and ELAV-GS Female Mortality Rates

Figure S2. Adult heads expression levels of CycD and Tfam mRNA

Figure S3. *Worn-Gal4* loss or gain of CycD/Cdk4 does not influence lifespan

Figure S4. DHE Superoxide indicator in *ELAV-GFP* CNS

Figure S5. Protein carbonyls and oxidative stress-dependent AGEs eliminated by feeding MitoTEMPO antioxidant. Mutants show no changes in modified protein carbonyls and oxidative stress-dependent AGEs.

Figure S6. Commassie staining of Westerns stained with DNPH or AGE.

Figure S7. Neuronal loss or gain of CycD/Cdk4 induces cell death

Figure S8. MitoTEMPO prevents neuronal death in loss or gain of CycD/Cdk4

Figure S9. Loss or gain of CycD/Cdk4 does not repress autophagy

Table S1: CycD/Cdk4 overexpression resembles Hyperoxia

Table S2: cycD mutants repress transcripts upregulated by Hyperoxia

Table S3: CycD/Cdk4 overexpression suppressed similar transcripts as Hyperoxia treatment

Table S4: cycD mutants upregulate transcripts suppressed by Hyperoxia treatment

Fly stocks and UAS transgenes

Additional Supplemental Methods.


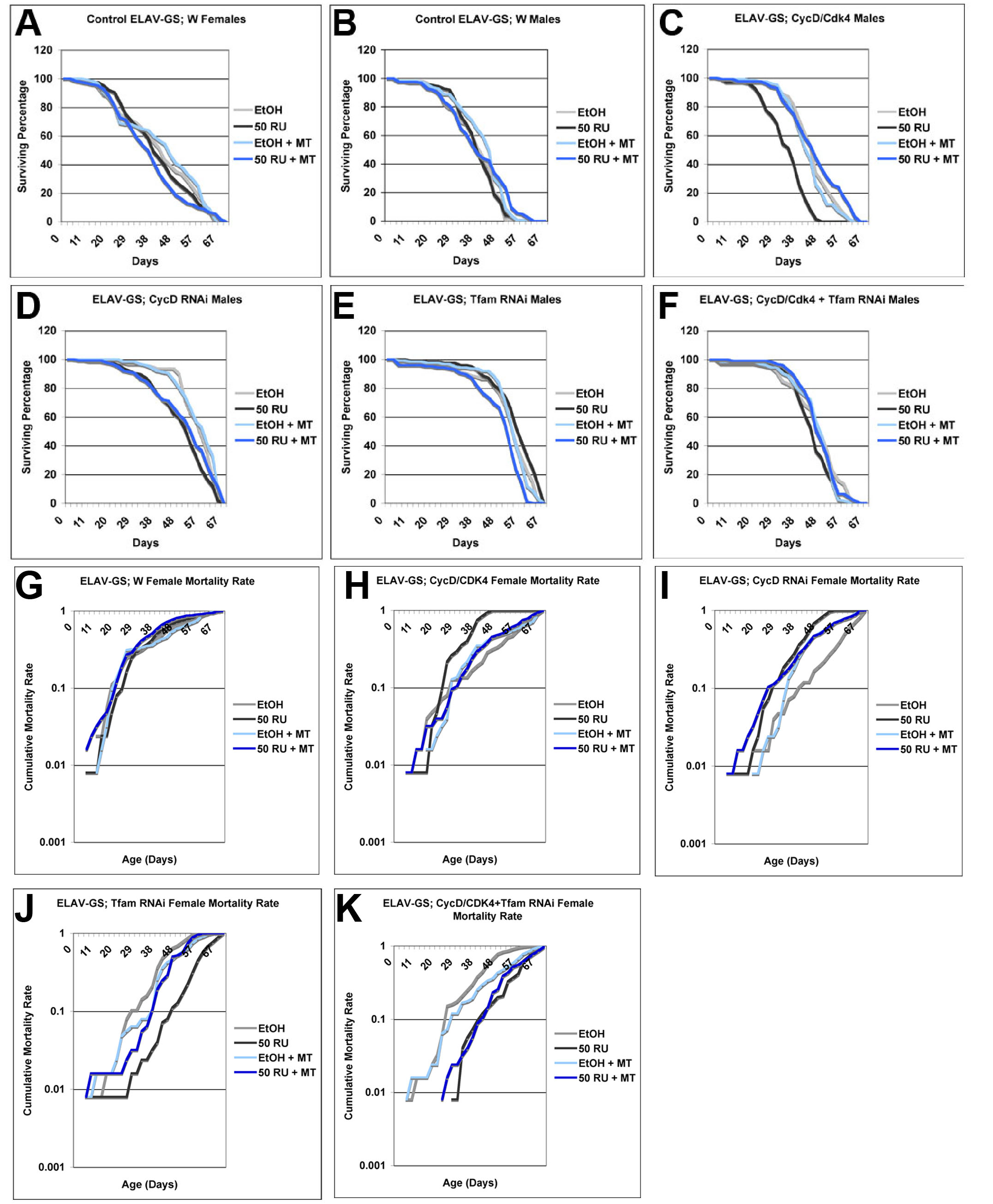


**Figure S1 Additional lifespan data.**

Lifespan in females and males with *ELAV-GeneSwitch*; UAS-X was analyzed. (A) Adult Females with ELAV-GeneSwitch; W^1118^ Control; (B) Adult males with ELAV-GeneSwitch; W^1118^ Control; (C) Adult males with ELAV-GeneSwitch; CycD/Cdk4 expression (EtOH vs. RU p=0; EtOH + MT vs. RU + MT p= 0.00181). (D) Adult males with ELAV-GeneSwitch; CycD RNAi knock-down (EtOH vs. RU p= 0.000179; EtOH + MT vs. RU + MT p= 0.0295). (E) Adult males with ELAV-GeneSwitch; Tfam RNAi knock-down (EtOH vs. RU p= 0.0976; EtOH + MT vs. RU + MT p= 9.93e-05). (F) Adult males with ELAV-GeneSwitch; CycD/Cdk4 expression + Tfam RNAi knock-down (EtOH vs. RU p= 0.00288; EtOH + MT vs. RU + MT p= 0.557). Motality Rate of (G) ELAV-GS; W Females, (H) ELAV-GS; CycD/Cdk4 Females, (I) ELAV-GS; CycD RNAi Females, (J) ELAV-GS; Tfam RNAi Females, (K) ELAV-GS; CycD/Cdk4 + Tfam RNAi Females.


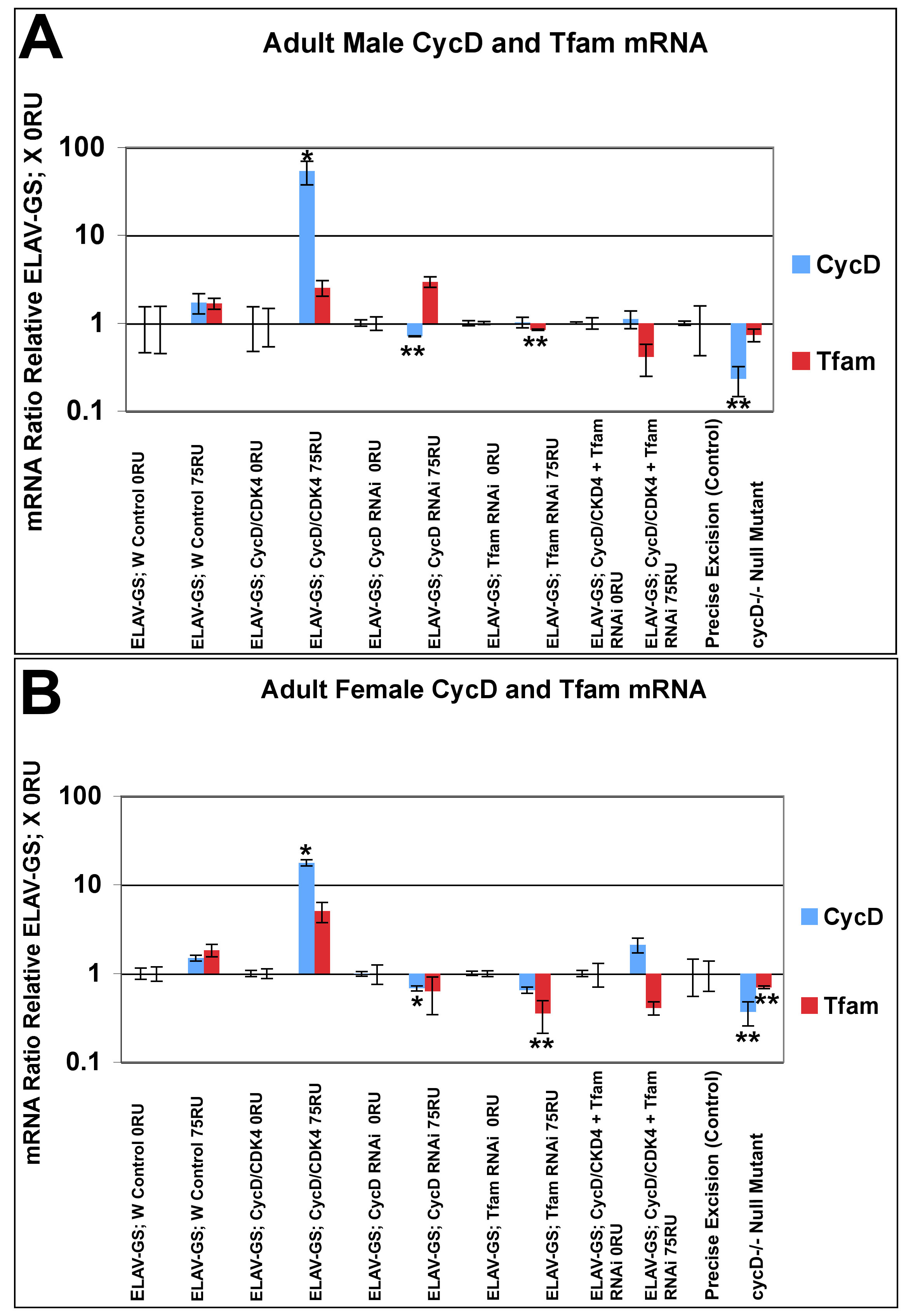


**Figure S2. Adult CNS expression of CycD and Tfam mRNA** (A) Adult Male CycD and Tfam mRNA of 4 day old (DO) adults of following genotypes: ELAV-GeneSwitch; W (control for ELAV-transgenes), with ELAV-GeneSwitch; ELAV-GeneSwitch, CycD/Cdk4; ELAV-GeneSwitch, CycD RNAi, ELAV-GeneSwitch, Tfam RNAi, ELAV-GeneSwitch, CycD/Cdk4 + Tfam RNAi, Precise Excision (control for cycD-/- mutant) and cycD-/- mutant. (B) Adult Female CycD and Tfam mRNA of 4 day old (DO) adults of following genotypes: ELAV-GeneSwitch; W (control for ELAV-transgenes), with ELAV-GeneSwitch; ELAV-GeneSwitch, CycD/Cdk4; ELAV-GeneSwitch, CycD RNAi, ELAV-GeneSwitch, Tfam RNAi, ELAV-GeneSwitch, CycD/Cdk4 + Tfam RNAi, and Precise Excision (control for cycD-/- mutant) and cycD-/- mutant. * p<0.05 ** p< 0.01.


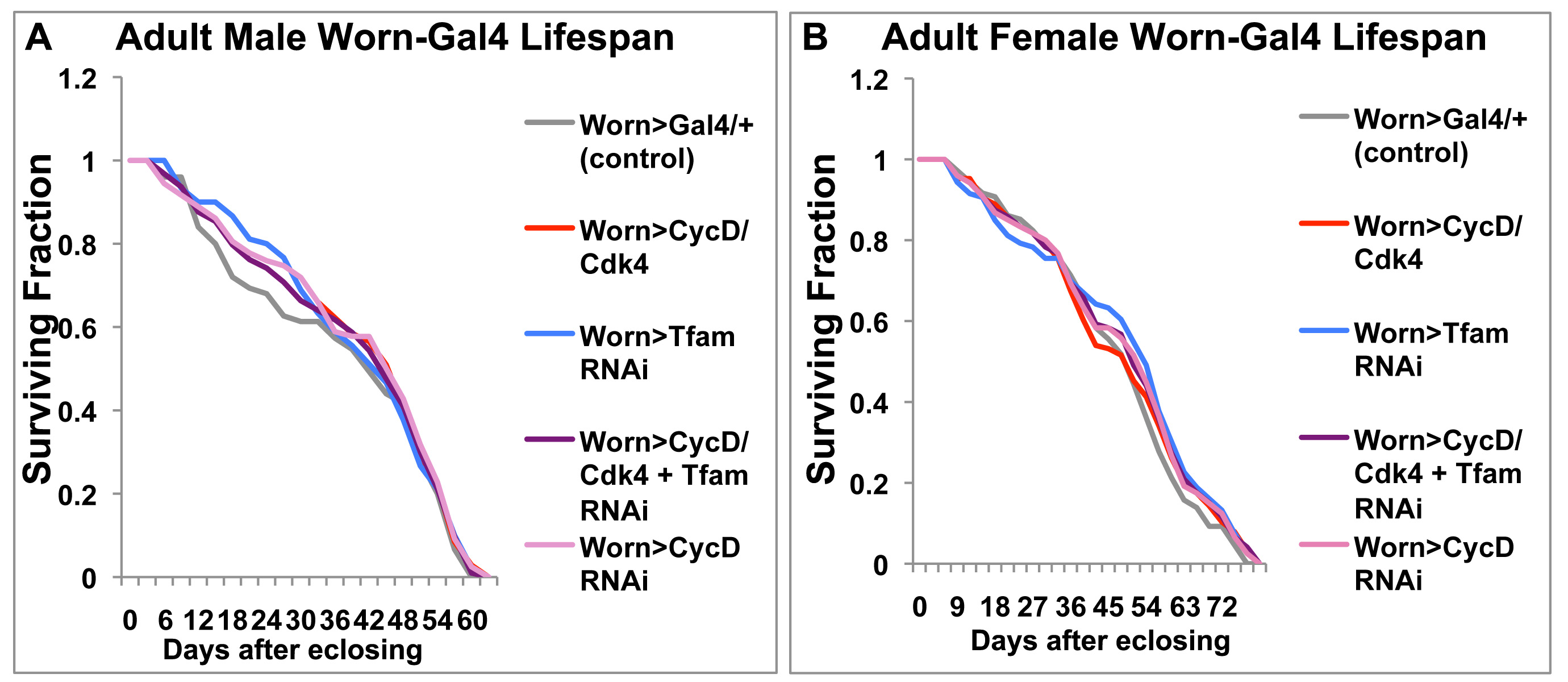


**Figure S3 Neuronal expression of CycD/Cdk4 via Worn-Gal4 does not influence adult lifespan.**

Lifespan in adults with *Worn-Gal4*/UAS-X was analyzed. (A) Adult males with Worn-Gal4 expression. (B) Adult females with Worn-Gal4 expression. No significant difference was found among genotypes.

**
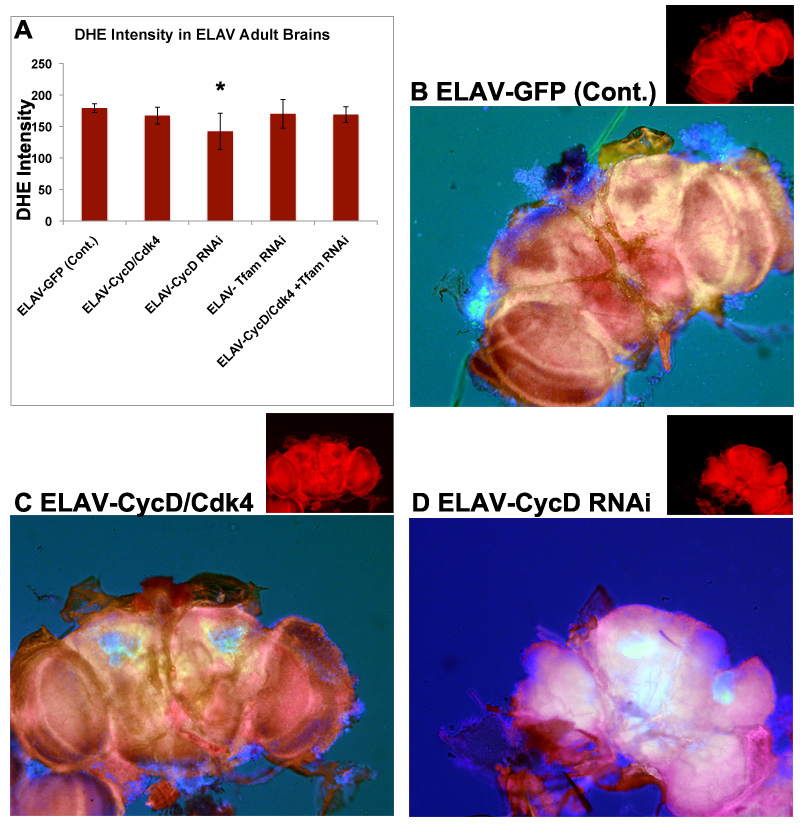
**

**Figure S4 Neuronal CycD/Cdk4 does not increase DHE**

DHE (cytoplasmic superoxide indicator) reagent in central nervous system cells expressing *ELAV-Gal4* driver marked with UAS-GFP. (A) Graph of quantification of DHE intensity of ELAV-GFP vs. control CNS (non-GPF) (B) ELAV-GFP (control), (C) CycD/Cdk4, (D) CycD RNAi, (E) Tfam RNAi, or (F) CycD/Cdk4 & Tfam RNAi. * indicates p<0.05. Methods from (Bahadora*ni et a*l. 2010b)


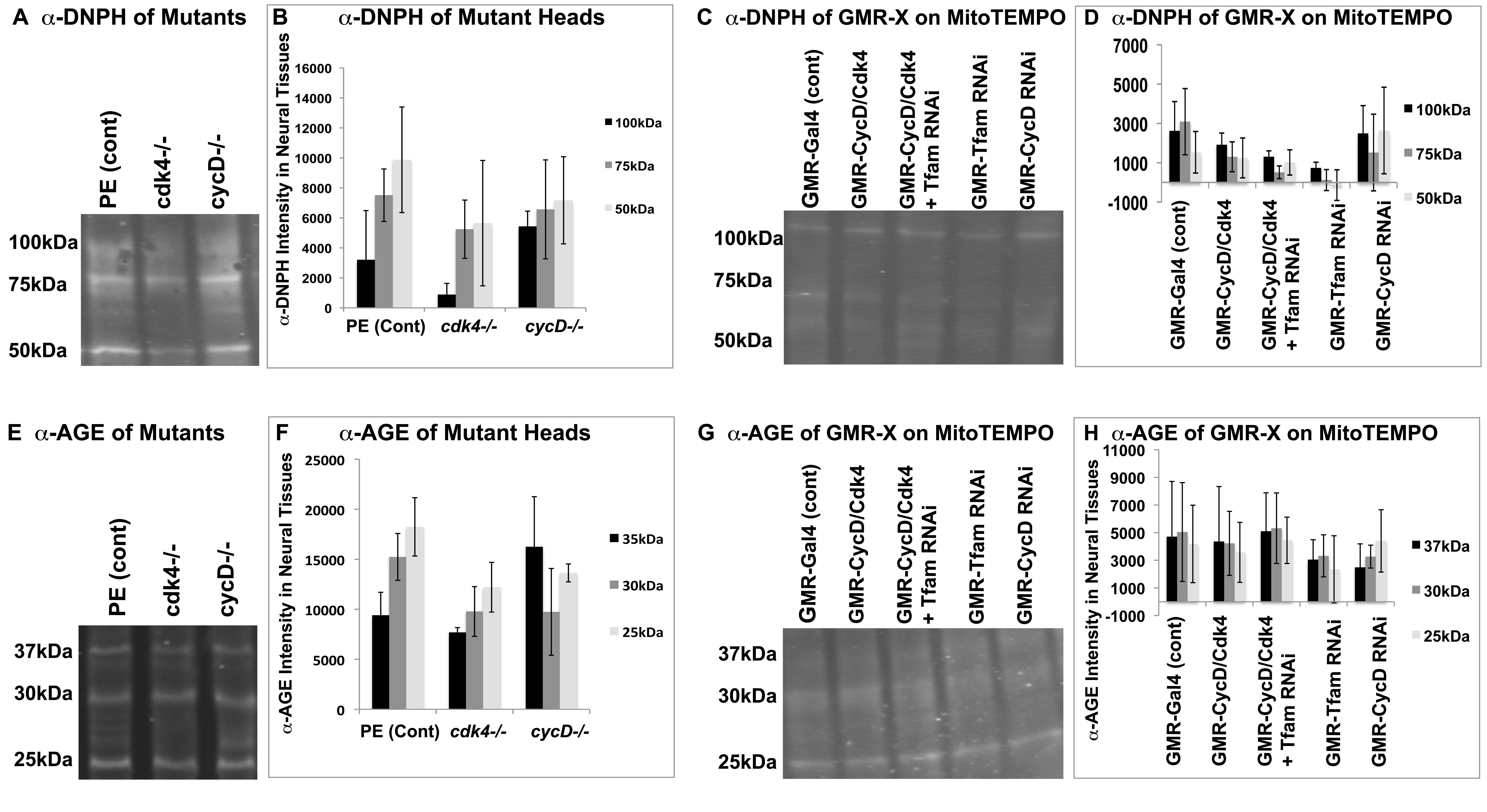


**Figure S5. Changes in protein carbonyls and oxidative stress dependent AGEs eliminated by MitoTEMPO antioxidant**

(A) Western bolt incubated with the OxiSelect Protein Carbonyl detection kit with genotypes listed above every lane. Four male heads at 7 days were used for each sample. (B) Graph of intensity quantification of three Western blots at three discrete bands of several kDa. (C) Western bolt incubated with the OxiSelect Protein Carbonyl detection kit with genotypes listed above every lane. Four male heads expressing *GMR-Gal4* for 7 days fed MitoTEMPO were used for each sample. (D) Graph of intensity quantification of three Western blots at three discrete bands of several kDa. Accumulation of advanced glycation end products (AGEs) are strongly associated with mortality rates of flies, and thus are classified as biomarkers of aging-related damage in Drosophila (Jacobs*on et a*l. 2010). (E) Image of western bolt incubated with anti-AGE antibodies, with genotypes listed above each lane. Four male heads were used for each sample. (F) Graph of intensity quantification of three Western blots at three discrete bands of several kDa. (G) Image of western bolt incubated with anti-AGE antibodies, with genotypes listed above each lane. Four male heads expressing *GMR-Gal4* for 7 days fed MitoTEMPO were used for each sample. (H) Graph of intensity quantification of three Western blots at three discrete bands of several kDa.

**Figure S6.** Coomassie stain of Westerns stained with either DNPH or AGE antibodies. Same area shown as regions and kDa bands measured for intensity of secondary antibody staining.


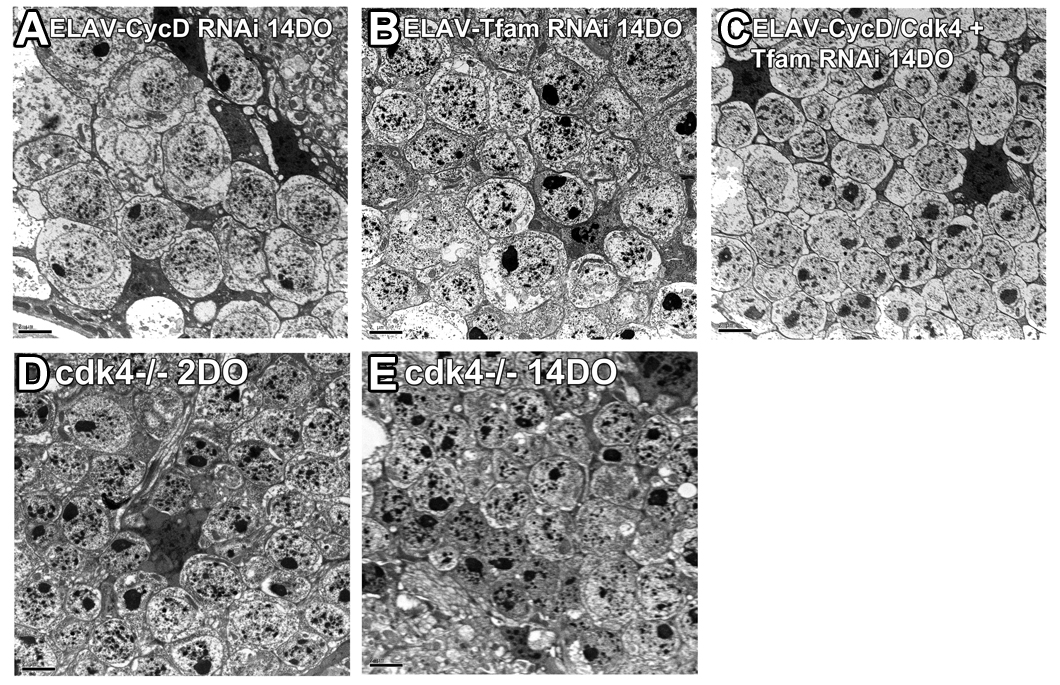


**Figure S7. Neuronal loss or gain of CycD/Cdk4 induces cell death**

(A) Adult male central nervous system expressing ELAV-CycD RNAi for 14 days (dead cells stain denser), (B) expressing ELAV-Tfam RNAi for 14 days, (C) adult male expressing ELAV-CycD/Cdk4 + Tfam RNAi 14 days, (D) cdk4-/- mutant adults at 2 days, (E) cdk4-/- mutants at 14 days. Quantification of cell death listed in main text.


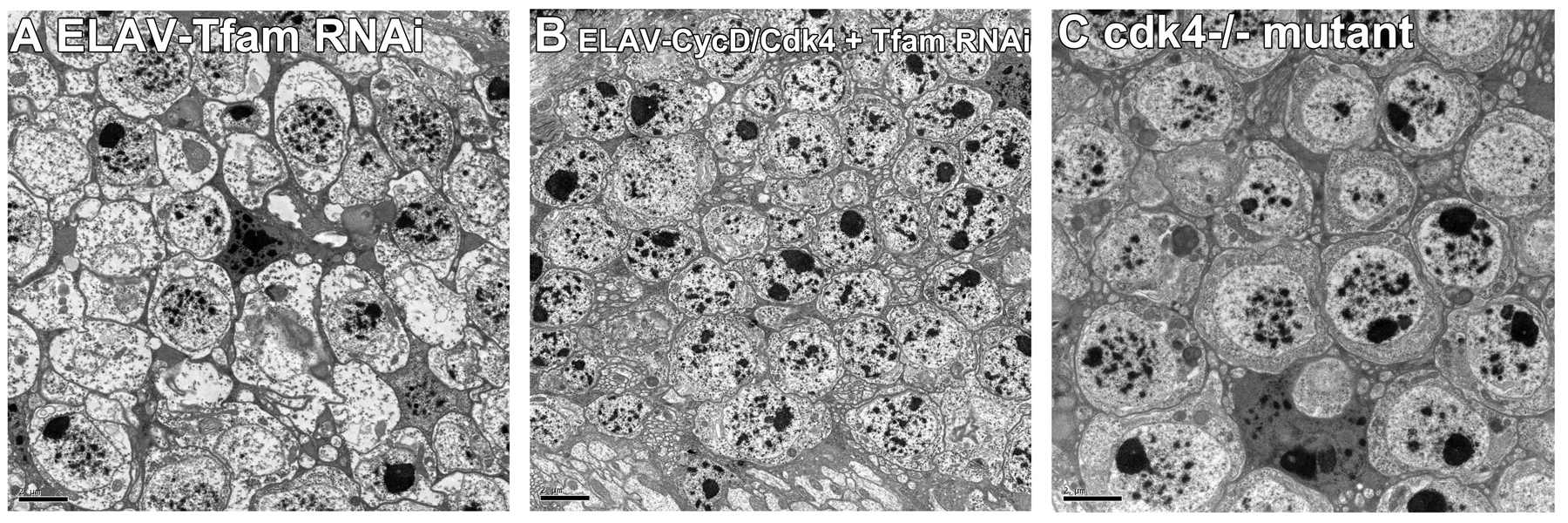


**Figure S8. MitoTEMPO prevents neuronal death in gain and loss of CycD or Cdk4**

Adult males fed MitoTEMPO, with (A) ELAV-Tfam RNAi for 14 days, (B) ELAV-CycD/Cdk4 + Tfam RNAi for 14 days, or (C) cdk4 mutants at 14 days. Quantification of cell death listed in main text.


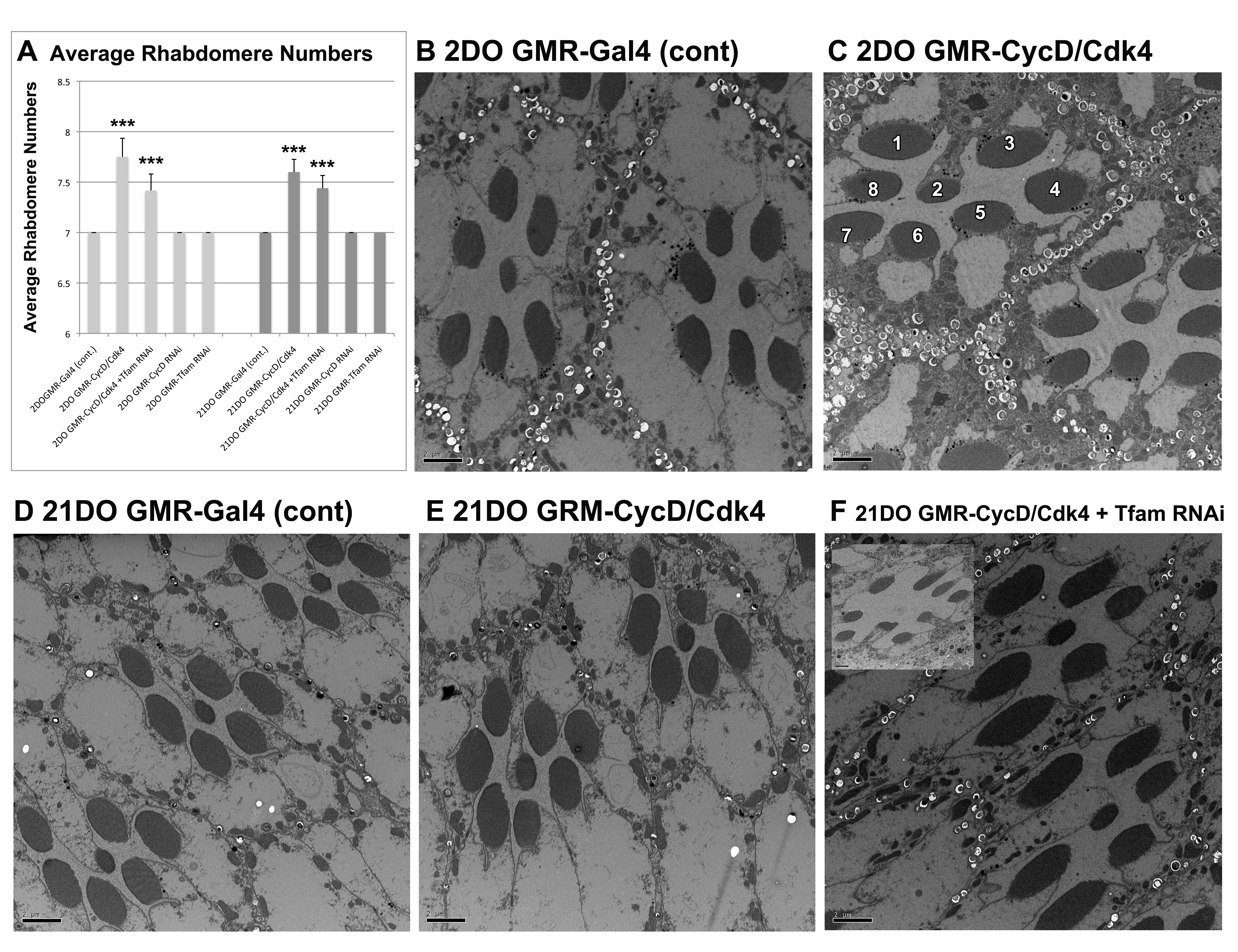


**Figure S9. GMR-CycD/Cdk4 does not induce neurodegeneration via autophagy**

Micrographs of adult male eyes indicating rhabdomeres per ommatidia of (A) Graph of the Mean # of Rhabdomeres per Ommatidium from 3 adult males; *** p<0.001. (B) GMR-Gal4 (control) at 2 days of overexpression (DO), (C) GMR-CycD/Cdk4 at 2DO, (D) GMR-Gal4 (control) at 21 DO, (E) GMR-CycD/Cdk4 at 21DO, and (F) GMR-CycD/Cdk4 + Tfam RNAi at 21DO. Methods from Wang et al. (Wa*ng et a*l. 2009).

**Supp. Table 1: CycD/Cdk4 overexpression resembles Hyperoxia**

CycD/Cdk4 significantly upregulates oxidative stress response; genes listed in red coincide with Gruenewald et al. hyperoxia-responsive transcripts, grey genes are involved in ROS neutralization (grey genes are not included in statistical analysis), (Gruenewa*ld et a*l. 2009). We also queried *in silico* for mitochondrial localization sequences (MLS -Yes) or secreted peptide sequence (S). *p < 1.57e-05.

| **Gene Name** | **Function** | **Fold Change in +CycD/ Cdk4 16h** | **Hyperoxia 100% O2 6D**  **(2009) Gruenewald et al.** | **100% oxygen & Old Flies (2004) Landis et al.** | **Paraquat & H2O2 oxidative stressors (2004) Girardot et al.** | **MLS?** |
| --- | --- | --- | --- | --- | --- | --- |
| CG8112 | Fatty acid metabolism | 2.19515 | 2 | 2 | 2.1 | N |
| ade3 | Purine metabolism | 3.18198 | 1.8 | 5.1 | 9.8 | S |
| l(3)02640 | Enzyme | 1.86988 | 1.8 | 1.9 | 1.8 | YES |
| RpA-70 | Nucleic acid binding | 1.72237 | 2.1 | 2.2 | 1.6 | N |
| CG2909 | Unknown | 1.59882 | 2.4 | 1.6 | 3.1 | N |
| Cyp309a1 | Detoxification/antioxidant | 1.71973 | 9.1 | 2.8 | 3.2 | N |
| Tsp42E | Signal transduction | 1.52737 | 2.3 | 2.6 | 2.3 | S |
| Cyp309a2 | Detoxification/antioxidant | 3.31026 | 4.2 | 1.8 | 2.3 | YES |
| CG10638 | Detoxification/antioxidant | 1.70965 | 4.9 | 1.7 | 1.5 | N |
| Arc2 | CG13941 | signal transduction | 1.50354 | 36.7 | 3.2 | 2.6 | N |
| Prx6005 | Glutathione (GSH) peroxidase activity | 1.71297 | NA | NA | NA | N |
| Cat | Catalase (CAT) | 1.67017 | NA | NA | NA | YES |

**Supp. Table 2. cycD mutants repress transcripts upregulated by Hyperoxia**

cycD mutants significantly repress oxidative stress response transcripts; genes listed in green coincide with Gruenewald et al. hyperoxia-responsive transcripts, grey genes are involved in ROS neutralization (grey genes are not included in statistical analysis) (Gruenewa*ld et a*l. 2009). p<0.0345.

| **Gene Name** | **Function** | **Fold Change in cycD mutants** | **Hyperoxia 100% O2 6D**  **(2009) Gruenewald et al.** | **100% oxygen & Old Flies (2004) Landis et al.** | **Paraquat & H2O2 oxidative stressors (2004) Girardot et al.** |
| --- | --- | --- | --- | --- | --- |
| Cyp309a1 | Detoxification/antioxidant | -3.117 | 9.1 | 2.8 | 3.2 |
| Cyp309a2 | Detoxification/antioxidant | -2.606 | 4.2 | 1.8 | 2.3 |
| CG11089 | Purine metabolism | -2.599 | 1.7 | 3.8 | 7.6 |
| CG6767 | Purine metabolism | -1.892 | 1.6 | 4.1 | 3.2 |
| CG3397 | Oxidoreductase | -1.845 | 1.6 | 2.1 | 2.5 |
| Prx2540-1 | Glutathione (GSH) peroxidase activity | -1.974 | NA | NA | NA |
| Cat | Catalase (CAT) | -1.682 | NA | NA | NA |

**Supp. Table 3. CycD/Cdk4 animals downregulate similar set of transcripts (28) as hyperoxia-adapted flies p< 2.74e-15.** Transcripts are culled from Zhao et al, wherein they hyperoxia-selected flies for many generations and then determined which genes were altered (Zh*ao et a*l. 2010). Gpdh, CG33129, and UGP single null mutants confer resistance to hyperoxia; CycD/Cdk4 expression transcriptionally represses all three.

| **Gene Symbol** | **Gene Title** | **Fold Change (Zhao et al.)** | **p-value (Zhao et al.)** | **Fold Change +CycD/Cdk4 for 16h** |
| --- | --- | --- | --- | --- |
| Gpdh | Glycerol 3 phosphate dehydrogenase | -2.047502048 | 2.1497E-14 | -1.5973675 |
| CG33129 | CG33129 | -1.756851722 | 5.9064E-10 | -2.6022015 |
| UGP | CG4347 | -2.225189141 | 1.3509E-05 | -1.8894662 |
| CG17633 | CG17633 | -1.680672269 | 7.8119E-09 | -2.2460806 |
| CG6271 /// CG6277 | CG6271 /// CG6277 | -2.359046945 | 6.6135E-19 | -2.0645374 |
| CG11911 | CG11911 | -1.685772084 | 7.4168E-09 | -2.158615 |
| CG10621 | selenocysteine methyltransferase | -1.940617116 | 9.9721E-13 | -1.8684953 |
| Ag5r2 | Antigen 5-related 2 | -1.635322976 | 5.383E-08 | -1.6911603 |
| Lsp1beta | Larval serum protein 1 beta | -4.020908725 | 6.1424E-34 | -1.5465034 |
| CG10531 | CG10531 | -2.014504432 | 1.58E-10 | -1.768159 |
| Cyp6a23 | Cyp6a23 | -1.862891207 | 3.5332E-09 | -2.7065064 |
| Act88F | arthrin | -6.234413965 | 2.9129E-46 | -1.5903814 |
| CG12057 | CG12057 | -8.058017728 | 1.7807E-51 | -1.7297148 |
| Lsd-1 | Lipid storage droplet-1 | -2.211900022 | 9.9937E-17 | -1.576392 |
| CG7532 | CG7532 | -1.723246597 | 4.4147E-09 | -1.6549989 |
| CG7214 | CG7214 | -5.06585613 | 2.5778E-42 | -2.5403277 |
| Lsp1beta | Larval serum protein 1 beta | -4.020908725 | 6.1424E-34 | -1.5420438 |
| CG7203 | CG7203 | -2.809778028 | 2.8265E-25 | -1.5983122 |
| CG7920 /// DsimCG7920 | CG7920 | -1.629195178 | 7.0415E-08 | -1.5888652 |
| Zeelin1 | CG6803 | -1.832844575 | 3.0822E-11 | -1.5100266 |
| Cpr49Ae | CG8505 | -2.283626399 | 5.6119E-18 | -1.5398355 |
| CG9466 | CG9466 | -1.639344262 | 8.383E-08 | -1.7818959 |
| CG9090 /// DsecCG9090 /// DsimCG9090 | CG9090 | -1.773678609 | 3.8196E-10 | -1.8880036 |
| up | troponin T | -1.684919966 | 1.3974E-08 | -1.5012986 |
| CG8630 | CG8630 | -2.054231717 | 1.3926E-09 | -1.7875657 |
| l(2)k05713 | mitochondrial GPD | -1.627869119 | 1.5115E-07 | -1.7528177 |

**Supp. Table 4. cycD mutants upregulate transcripts downregulated by hyperoxia-adapted flies p<1.811e-07.** Transcripts are culled from Zhao et al, wherein they hyperoxia-selected flies for many generations and then determined which genes were altered (Zh*ao et a*l. 2010). Gpdh single null mutants confer resistance to hyperoxia; cycD mutants transcriptionally upregulate Gpdh.

| **Gene Symbol** | **Gene Title** | **Fold Change (Zhao et al.)** | **p-value (Zhao et al.)** | **Fold Change in cycD mutants** |
| --- | --- | --- | --- | --- |
| Gpdh | Glycerol 3 phosphate dehydrogenase | -2.048 | 2.15E-14 | 2.08462 |
| regucalcin | Regucalcin | -2.481 | 2.75E-19 | 49.69732 |
| Ag5r2 | Antigen 5-related 2 | -1.635 | 5.38E-08 | 4.12569 |
| Scp2 | Calexcitin | -1.623 | 9.71E-08 | 4.89603 |
| CG8012 | CG8012 | -1.603 | 4.24E-07 | 5.26554 |
| CG7532 | CG7532 | -1.723 | 4.41E-09 | 2.91004 |
| Obp56a | Odorant-binding protein 56a | -1.820 | 6.37E-10 | 2.50244 |
| CG3290 | CG3290 | -1.862 | 5.67E-09 | 2.45671 |
| Cyp6a23 | Cyp6a23 | -1.863 | 3.53E-09 | 2.45147 |
| CG10621 | selenocysteine methyltransferase | -1.941 | 9.97E-13 | 2.25468 |
| CG5150 | CG5150 | -2.047 | 2.98E-12 | 2.11510 |
| Pdh | pigment cell dehydrogenase reductase | -2.578 | 1.54E-21 | 3.77616 |
| CG31775 | CG31775 | -3.293 | 1.97E-21 | 2.36068 |
| Lsp1beta | Larval serum protein 1 beta | -4.021 | 6.14E-34 | 2.22616 |
| CG7214 | CG7214 | -5.066 | 2.58E-42 | 2.12139 |
| CG12057 | CG12057 | -8.058 | 1.78E-51 | 2.08701 |

**Fly stocks and UAS transgenes**

PE; +; + (Precise excision-cycD-/- control); (Emmerich, 2004)

w; cdk4^3^/CyO-GFP; + (Meyer et al., 2000)

cycD^1^/FM7; +; + (Emmerich, 2004)

w^1118^; +; + (Bloomington Stock 5905)

w; UAS-CycD, UAS-Cdk4; + (UAS-CycD/Cdk4); (Datar et al., 2000)

w; UAS-CycD RNAi (KK); + (VDRC Stock 105361)

w; UAS-Tfam RNAi/CyO; + (Kyoto Stock 4217R-2)

w; UAS-CycD/Cdk4; UAS-Tfam RNAi; (Datar et al., 2000; combined with Kyoto Stock 4217R-1)

ELAV-Gal4, UAS-GFP/ FM7C; +; + (Bloomington Stock 23867)

yw, ELAV-GeneSwitch (Bloomington Stock 43642)

GMR-Gal4/CyO; (Bloomington Stock 9146)

Worn-Gal4; +; + (Gift from Dr. Alexandre Neves, Dr. Robert Eisenman’s Lab, FHCRC)

**TEM (for autophagy analysis)**

Six heads from 2 and 21 day old GMR-Gal4, UAS-X males were were fixed o/n at 4°C in 2% PFA plus 4% glutaraldehyde, postfixed in 1% osmium tetroxide (OsO_4_) at RT, dehydrated in an ethanol series, and embedded in LR White. Ultrathin sections (80 nm) were examined with JEOL 1230 transmission electron microscope at 100 kV. 100 micrographs from each genotype were taken at 1,000X magnification, similar to (Wa*ng et a*l. 2009).

**DHE**

Fly brains were dissected in PBS buffer) and stained with 30 μM DHE solution for 10-minutes. Brains were washed 3 times in PBS buffer and visualized with Nikon Ti microscope (similar to (Bahadora*ni et a*l. 2010a; Bahadora*ni et a*l. 2010b). The intensity of the brains was measured using the ImageJ software to measure mean pixel intensity.

**mRNA Primer Sequences**

CG5999

CG5999F GAGCTATTCTCCTCTCCCTG

CG5999R TCCCACTTCCAAATCACCC

CycD

CycD1F TCGAGCATAGCTGCGTCAAT

CycD1R CTTGCTCCACGCTGGTTAGA

CycD2F ATCAGTACGCAAGACCCAGC

CycD2R GATCCACCGAAAGAGCACGA

Cyp6d2F tcccgtctattttcccaatccc

Cyp6d2R aaatcttagccaccgccacc

Cyp309a1

Cyp309a1F ccacgaagagcaggaaaag

Cyp309a1R gaaaccaatgggagtagtcag

Dp (internal control)

DpF 5' AGGTACCACCTACACCACCG

DpR 5' GTGCAACGAGTTGCTCTGAA

Hsp22

Hsp22F cccacaaaactttctctctcc

Hsp22R cactgttctttagcgaactcc

Sni

sniF tctgtatccgcaacgcatc

sniR aaccgccgttctgtttctc

Tfam

Tfam1F CGACTCCGAGAAGGAGGTCT

Tfam1R AAGATTTCCGTGACGCACCA

Tfam2F CCAAAAGACCACCGCCAAGT

Tfam2R GTAGAGCTCCATCTCCTTGCG

**References:**

Bahadorani S, Bahadorani P, Marcon E, Walker DW , Hilliker AJ (2010a). A Drosophila model of Menkes disease reveals a role for DmATP7 in copper absorption and neurodevelopment. *Dis Model Mech*. **3**, 84-91.

Bahadorani S, Cho J, Lo T, Contreras H, Lawal HO, Krantz DE, Bradley TJ , Walker DW (2010b). Neuronal expression of a single-subunit yeast NADH-ubiquinone oxidoreductase (Ndi1) extends Drosophila lifespan. *Aging Cell*. **9**, 191-202.

Gruenewald C, Botella JA, Bayersdorfer F, Navarro JA , Schneuwly S (2009). Hyperoxia-induced neurodegeneration as a tool to identify neuroprotective genes in Drosophila melanogaster. *Free Radic Biol Med*. **46**, 1668-1676.

Wang T, Lao U , Edgar BA (2009). TOR-mediated autophagy regulates cell death in Drosophila neurodegenerative disease. *J Cell Biol*. **186**, 703-711.

Zhao HW, Zhou D, Nizet V , Haddad GG (2010). Experimental selection for Drosophila survival in extremely high O2 environments. *PLoS One*. **5**, e11701.
